# Modest vasomotor dysfunction induced by low doses of C_60 _fullerenes in apolipoprotein E knockout mice with different degree of atherosclerosis

**DOI:** 10.1186/1743-8977-6-5

**Published:** 2009-02-25

**Authors:** Lise K Vesterdal, Janne K Folkmann, Nicklas R Jacobsen, Majid Sheykhzade, Håkan Wallin, Steffen Loft, Peter Møller

**Affiliations:** 1 Department of Public Health, Section of Environment Health, University of Copenhagen, Øster Farimagsgade 5A, DK-1014 Copenhagen K, Denmark; 2 Danish Research Centre for the Working Environment, Lersø Parkallé 105, DK-2100 Copenhagen Ø, Denmark; 3 Department of Pharmacology and Pharmacotherapy, Faculty of Pharmaceutical Sciences, University of Copenhagen, Universitetsparken 2, DK-2100 Copenhagen Ø, Denmark

## Abstract

**Background:**

Exposure to small size particulate matter in urban air is regarded as a risk factor for cardiovascular effects, whereas there is little information about the impact on the cardiovascular system by exposure to pure carbonaceous materials in the nano-size range. C_60 _fullerenes are nano-sized particles that are expected to have a widespread use, including cosmetics and medicines.

**Methods:**

We investigated the association between intraperitoneal injection of pristine C_60 _fullerenes and vasomotor dysfunction in the aorta of 11–13 and 40–42 weeks old apolipoprotein E knockout mice (apoE^-/-^) with different degree of atherosclerosis.

**Results:**

The aged apoE^-/-^mice had lower endothelium-dependent vasorelaxation elicited by acetylcholine in aorta segments mounted in myographs and the phenylephrine-dependent vasoconstriction response was increased. One hour after an intraperitoneal injection of 0.05 or 0.5 mg/kg of C_60 _fullerenes, the young apoE^-/- ^mice had slightly reduced maximal endothelium-dependent vasorelaxation. A similar tendency was observed in the old apoE^-/- ^mice. Hampered endothelium-independent vasorelaxation was also observed as slightly increased EC_50 _of sodium nitroprusside-induced vasorelaxation response in young apoE^-/- ^mice.

**Conclusion:**

Treatment with C_60 _fullerenes affected mainly the response to vasorelaxation in young apoE^-/- ^mice, whereas the vasomotor dysfunction in old apoE^-/- ^mice with more advanced atherosclerosis was less affected by acute C_60 _fullerene treatment. These findings represent an important step in the hazard characterization of C_60 _fullerenes by showing that intraperitoneal administration is associated with a moderate decrease in the vascular function of mice with atherosclerosis.

## Background

The increasing use of nanomaterials warrants a thorough understanding of the hazards of the environment and man [1]. C_60 _fullerenes are spherical molecules with a diameter of around 1 nm, comprising 60 carbon atoms arranged in a truncated icosahedron structure similar to a soccer ball. C_60 _fullerenes have already been marketed in cosmetics, whereas their cage like structures are being developed e.g. for drug delivery with surface modification for targeting properties including cancer and antimicrobial activity. Multiple other applications can be envisaged, although widespread commercial use of C_60 _fullerenes is still in the future [2].

In keeping with the notion that exposure to particulate matter in air pollution is associated with increased risk of cardiovascular diseases, engineered nanomaterials are expected to generate similar effects [3]. The detrimental effects on the cardiovascular system of particulate matter in the small size range include altered activity of the fibrinolytic system, a procoagulant state, endothelial dysfunction, and cardiac effects [3-5]. Of special interest is the studies showing that airway exposure to concentrated ambient particles and single wall carbon nanotubes can promote progression of the atherosclerosis process in apolipoprotein E knockout mice (apoE^-/-^) that develop plaques in blood vessels at early age [6-8]. Similarly, pulmonary exposure to nano-sized carbon black is associated with accelerated plaque area development in the aorta of low-density lipoprotein receptor knockout mice that are also predisposed to atherosclerosis [9]. In addition, studies relating exposure to particulate matter by exposure to air pollution or diesel exhaust particles have shown increased area of atherosclerotic lesions in the aorta of Watanabe heritable hyperlipidemic rabbits [10,11].

The endothelium plays an important role in maintaining the vascular homeostasis by producing vasoactive factors that regulate the tone of the vascular system in response to cell surface receptor stimulation or mechanical stress [12]. In the functional endothelium, acetylcholine (ACh) binding to muscarinic receptors on the luminal surface is associated with the generation of nitric oxide (NO) by endothelial nitric oxide synthase (eNOS). The NO subsequently diffuses into the smooth muscle cells and facilitates the relaxation of the vessel. In experimental settings, the endothelium-independent vasorelaxation can be studied by NO-donors such as sodium nitroprusside (SNP). In contrast, the phenylephrine (PE) mediated vasoconstriction is mediated by binding to α_1_-adrenoceptors on the smooth muscle cells. Endothelial dysfunction is believed to be an event that leads to atherosclerosis as well as being a marker of the severity of atherosclerosis [13]. In apoE^-/- ^mice, the vasomotor function is inversely correlated with the plaque-size, whereas it is not affected by hypercholesterolemia [14]. In apoE^-/- ^mice, the acetylcholine-induced vasorelaxation was decreased and the PE-induced maximal vasoconstriction was enhanced after 5 months inhalation exposure to concentrated air pollution particles although this could be due to much further progressed aortic plaque development [6,8]. A number of studies have shown acute effects of particle exposure on vascular functions. E.g. ultrafine TiO_2 _particles were more potent in inducing systemic microvascular dysfunction in rats after exposure than fine TiO_2 _particles after inhalation at doses with minimum pulmonary effects [15]. We have shown that systemic exposure to diesel exhaust particles by intraperitoneal (i.p.) injection reduced acetylcholine-elicited vasorelaxation in apoE^-/- ^mice with minimum atherosclerosis, whereas opposite effects were seen in wild type mice [16].

Research concerning the effect of nanomaterials on the vascular function is still very sparse. The effect of C_60 _fullerenes or derivatives on vascular function has only been investigated in a few studies and only in *ex vivo *exposure designs. It has been shown that the endothelium-dependent relaxation of rabbit thoracic aorta segments is affected by water-soluble monomalonic acid-modified C_60 _fullerenes, whereas the PE-induced vasoconstriction was unaffected [17,18]. Water-soluble C_60_(OH)_24 _fullerenes have been shown to induce the endocytotic uptake of oxidized low-density-lipoproteins in macrophages and stimulate ADP-induced platelet aggregation [19]. To the best of our knowledge no data on vasomotor function following *in vivo *exposure to C_60 _fullerenes have been published.

The purpose of this study was to investigate the effect of systemic exposure to pristine C_60 _fullerenes by i.p. injection on the vascular function in apoE^-/- ^mice with varying degree of atherosclerosis. I.p. injection of C_60 _fullerenes has been used in some investigations as a route of exposure in order to focus on C_60 _fullerene's ability to prevent toxic effects of carbon tetrachloride [20] and methamphetamine and morphine [21]. We used the i.p. route of exposure and 1 hour post-treatment time similarly to our previous study on diesel exhaust particles in order to investigate direct systemic vascular effects unrelated to inflammatory responses [16]. The i.p. route allows a relatively slow systemic dose rate which would be difficult to achieve with i.v. injection and it is also similar to intramuscular or subcutaneous administration or even dermal uptake or translocation from the airways. We hypothesized that exposure to C_60 _fullerenes would affect the vasomotor function in aorta vessels with atherosclerosis in a similar way as diesel exhaust particles are associated with microvascular dysfunction.

## Results

### Characteristics of atherosclerosis and vasomotor function in apoE^-/- ^mice

**Figure 1:**
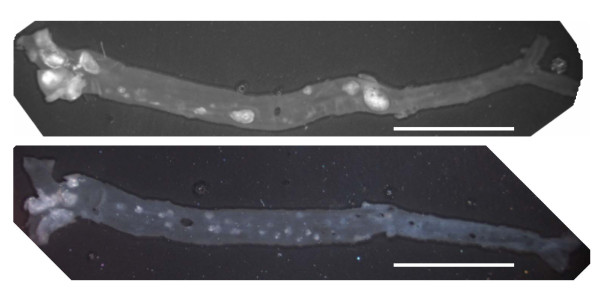
**Digital microscope images of the intimal surface of the aorta from the ascending aorta to the femoral arteries, dissected from two 40–42 weeks old female apoE^-/- ^mice**. The atherosclerotic plaques are discernible as white areas of the light grey background of normal aorta. The plaque area was determined by means of digital microscope images on which the plaque-area was determined with a Leica imaging program IM50 (Leica Microsystems Imaging Solutions). The scale bars represent 1 cm.

We used apoE^-/- ^mice of different age in order to test mice with different extents of aortic plaque development. In two mice aged 40–42 weeks the plaque area was assessed (figure 1). The plaque area in the aortic arch was 52% and 66%, whereas in the more distal part of the aorta the plaques comprised an area of 4% and 8%, respectively. In the whole aorta the plaques covered an area of 12% and 19% in the two mice, respectively.

**Figure 2:**
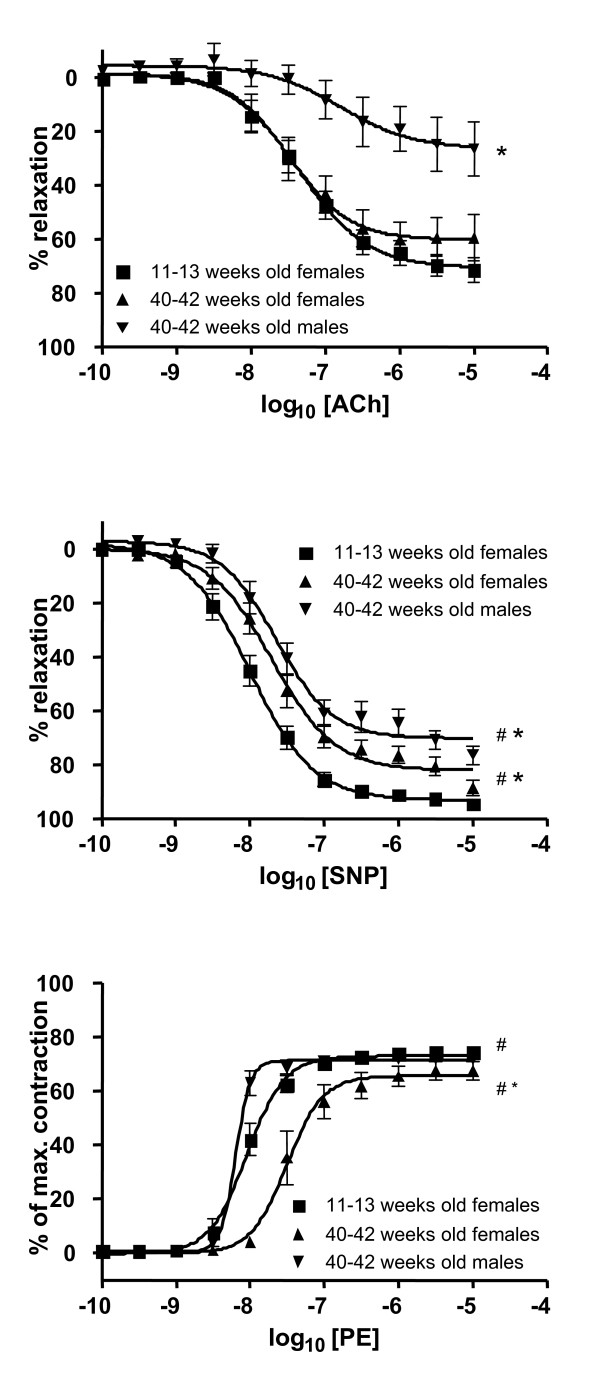
**Vascular function in aorta segments from the control groups of young and old apoE^-/- ^mice**. The aorta segments originated from the aortic arch and descending aorta in the young and old female mice, respectively. In the old male apoE^-/- ^mice, the vascular function was analyzed in aorta rings from the arch. ^# ^and * denotes statistically significant differences in EC_50 _and E_max_, respectively, observed for the aged control groups as compared with the young control group (P < 0.05, ANOVA with unequal variance between groups).

The primary aim of the study was to investigate the effect of C_60 _fullerenes at different stages of atherosclerosis by using apoE^-/- ^mice with different age. We carried out a small pilot study on 40–42 weeks old male apoE^-/- ^mice in order to assess whether or not it was possible to measure particle-induced differences in the vascular function in aorta segments highly covered with atherosclerotic plaques. This pilot study indeed indicated that especially the endothelium-dependent dysfunction in the segments of aortic arch from aged male mice was severely affected (figure 2).

### Effect of C_60 _fullerenes on the vasomotor function

#### Endothelium-dependent vasorelaxation

**Figure 3:**
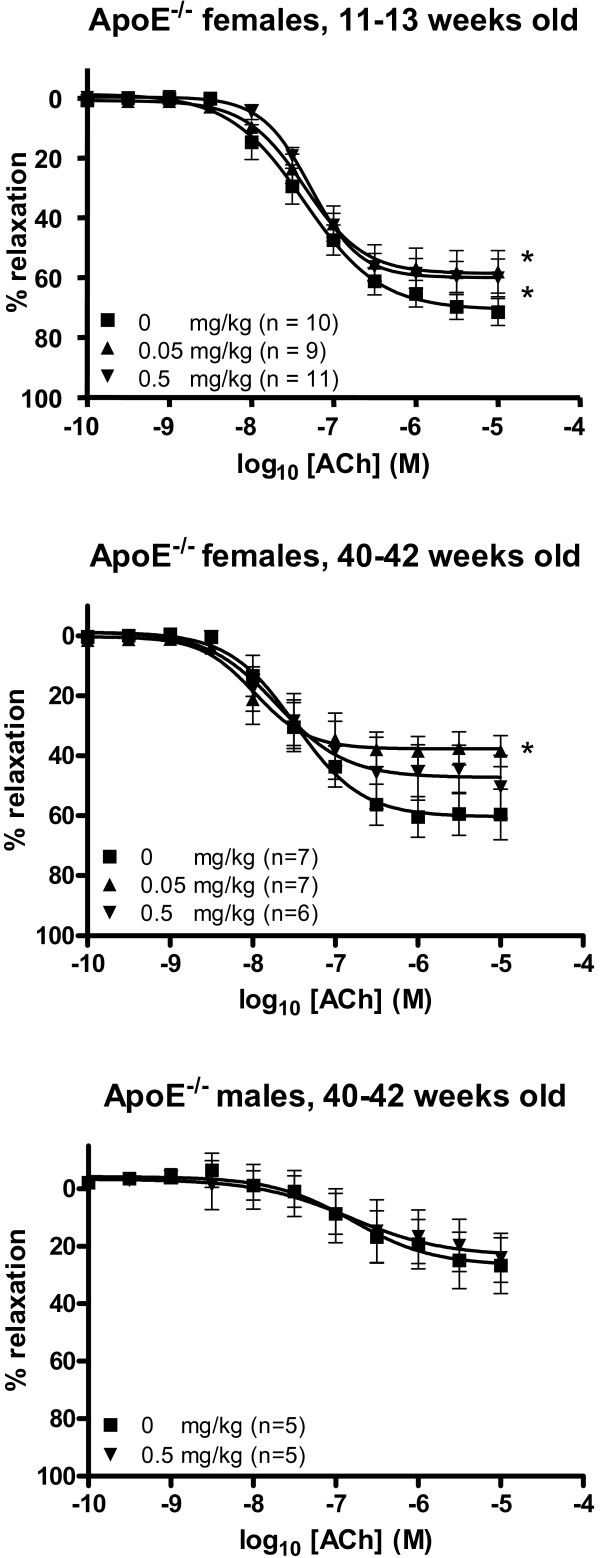
**Endothelium-dependent vasorelaxation of aorta segments from apoE^-/- ^mice treated with C_60 _fullerenes by i.p. injection**. The response is expressed as the percent relaxation of the precontraction tension produced by prostaglandin 2α. Each point on the curves represents the cumulative response at each concentration of acetylcholine (ACh). The data are expressed as the mean and SEM. * denotes a significant effect on E_max _compared to the control group (P < 0.05, ANOVA with unequal vaiance between groups).

The E_max _values of the acetylcholine-dependent vasorelaxation were significantly lower for both the low and the high dose of C_60 _fullerene treated female mice than for the controls (58.7% (95% CI: 52.9–64.4%) and 60.0% (95% CI: 55.9–64.2%) versus 70.8% (95% CI: 65.0–76.5%), respectively; figure 3, table 1). A similar pattern was observed in the old female mice; the E_max _value was significantly lower in the group of mice that had received 0.05 mg/kg C_60 _fullerenes (37.8% (95% CI: 32.6–43.0%)) compared to the control group (60.4% (95% CI: 53.2–67.6%)), whereas the E_max _value for the high dose exposed group was reduced (47.3% (95% CI: 38.6–56.0%)) although the difference was not quite statistically significant at a 5% level. There was no effect of C_60 _fullerene treatment in the old male apoE^-/- ^mice, which is likely to be because of the pre-existing poor vasomotor function in the segments obtained from these mice.

**Table 1:** EC_50 _and E_max _values of concentration-response curves performed for phenylephrine (PE), acetylcholine (ACh) and sodium nitroprusside (SNP) on 11–13 weeks female (F), 40–42 weeks female (F) and 40–42 weeks male (M) apoE^-/- ^mice.

**Dose of C_60 _fullerenes**	**EC_50 _(nM)**			**E_max _(%)**		
		
	**11–13 weeks (F)**	**40–42 weeks (F)**	**40–42 weeks (M)**	**11–13 weeks (F)**	**40–42 weeks (F)**	**40–42 weeks (M)**
*Vasoconstriction (PE)*						
0.00 mg/kg	9.0 (7.6–10.8)	31.4 (23.6–41.7)^¤^	6.4 (5.5–7.3)^¤^	72.9 (70.4–75.4)	65.6 (61.2–69.9)^¤^	71.3 (69.8–72.7)
0.05 mg/kg	8.7 (6.8–11.1)	31.7 (25.2–39.8)	ND	72.4 (69.1–75.6)	71.1 (67.2–75.1)	ND
0.50 mg/kg	8.1 (6.6–10.0)	38.9 (33.7–44.9)	6.8 (5.3–8.7)	73.7 (70.9–76.4)	69.5 (67.0–72.0)	67.0 (64.3–69.8)*
*Endothelium-dependent vasorelaxation (Ach)*						
0.00 mg/kg	44.3 (29.3–66.9)	32.7 (17.6–66.6)	161.6 (25.8–1022)	70.8 (65.0–76.5)	60.4 (53.2–67.6)	26.7 (13.0–40.4)^¤^
0.05 mg/kg	44.7 (27.4–72.8)	10.5 (4.7–23.4)	ND	58.7 (52.9–64.4)*	37.8 (32.6–43.0)*	ND
0.50 mg/kg	52.6 (38.6–71.8)	19.7 (6.9–56.1)	148.4 (7.7–2847)	60.0 (55.9–64.2)*	47.3 (38.6–56.0)	34.4 (4.9–41.9)
*Endothelium-independent vasorelaxation (SNP)*						
0.00 mg/kg	10.1 (8.1–12.6)	19.9 (14.4–27.4)^¤^	22.9 (15.8–33.1)^¤^	93.2 (90.1–96.3)	82.3 (77.8–86.8)^¤^	70.3 (65.4–75.2)^¤^
0.05 mg/kg	15.7 (11.2–22.0)	13.6 (8.2–22.5)	ND	86.6 (81.9–91.2)	80.2 (73.7–86.7)	ND
0.50 mg/kg	16.5 (13.4–20.3)*	20.9 (16.3–26.8)	20.3 (10.6–39.2)	88.3 (85.1–91.6)	83.3 (79.6–87.0)	58.5 (52.2–66.8)

The data are expressed as mean and 95% CI. The vasomotor function in the group of 0.05 mg/kg bodyweight was not determined (ND) in the male mice. * denotes statistically significant effects on EC_50 _or E_max _as compared to the effect in the control group (P < 0.05, ANOVA with unequal variance between groups). ^¤ ^denotes statistically significant differences in EC_50 _or E_max_, observed for the aged control groups as compared with the young control group (P < 0.05, ANOVA with unequal variance between groups).

#### Endothelium-independent vasorelaxation

**Figure 4:**
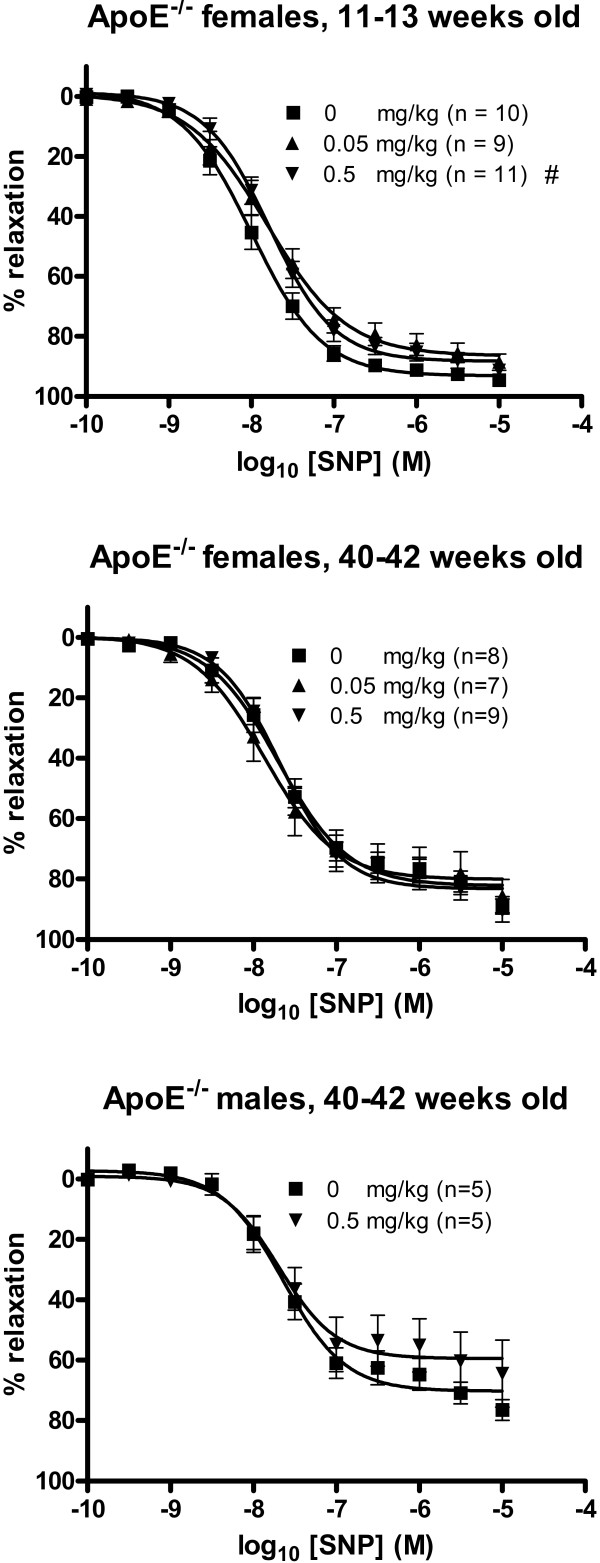
**Endothelium-independent relaxation of aorta segments from apoE^-/- ^mice treated with C_60 _fullerenes by i.p. injection**. The response is expressed as the percent relaxation of the precontraction tension produced by PGF_2α_. Each point on the curve represents the cumulative response at each concentration of sodium nitroprusside (SNP). The data are expressed as the mean and SEM. ^# ^denotes a significant effect on EC_50 _compared to the control group (P < 0.05, ANOVA with unequal variance between groups).

The SNP-induced vasorelaxation was shifted toward a reduced responsiveness in young apoE^-/- ^mice treated with 0.5 mg/kg of C_60 _fullerenes compared with the 0 mg/kg exposed group (figure 4, table 1). This was evident from the EC_50 _value (16.5 nM (95% CI: 13.4–20.3 nM) which was significantly increased compared to the control group (10.1 nM (95% CI: 8.1–12.6 nM). The EC_50 _for mice treated with the lower dose was slightly increased (15.7 nM (95% CI: 11.2–22.0 nM), although this was not statistically significant at a 5% level. The EC_50 _values for the low or high dose exposed old mice of both sexes exhibited no significant differences from the corresponding control values. For all groups of mice, there was no statistically significant effect of C_60 _fullerenes on the SNP-induced maximal endothelium independent vasorelaxation (E_max_), although the small group of old male mice appeared to show a decrease in E_max_.

#### Receptor-dependent vasoconstriction

**Figure 5:**
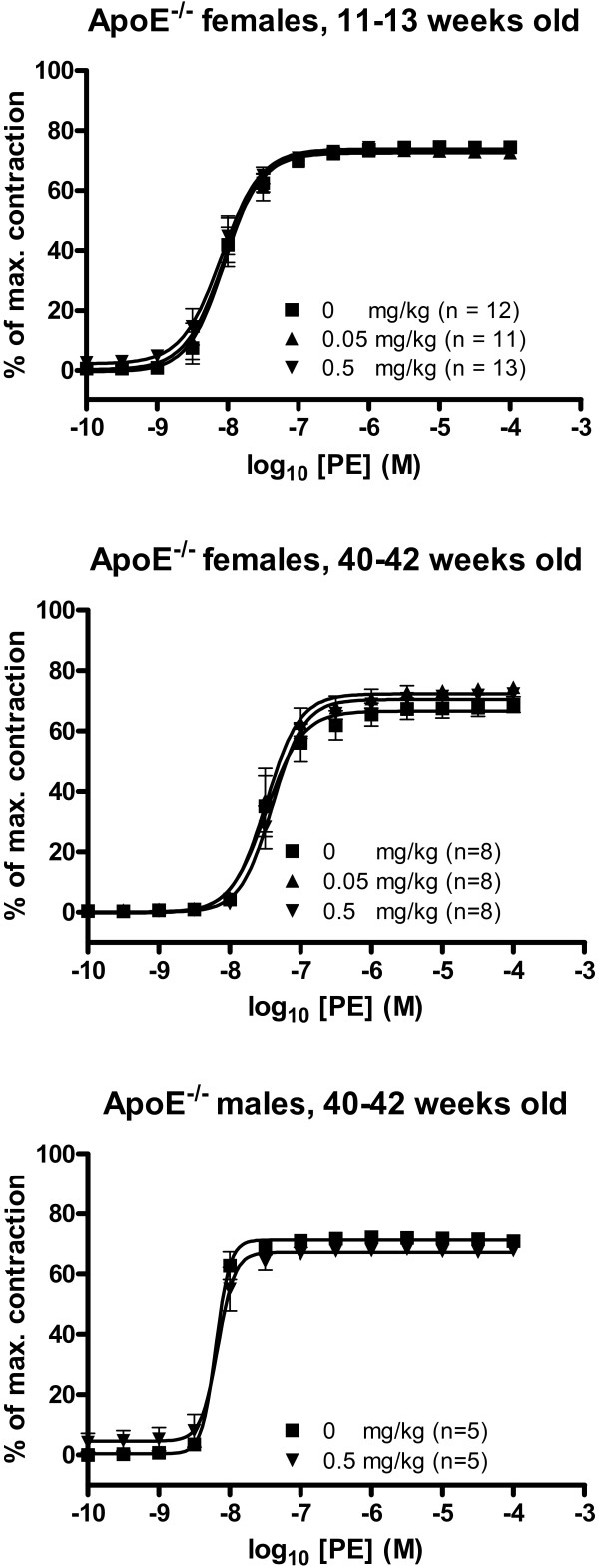
**Receptor-dependent vasoconstriction of aorta segments from apoE^-/- ^mice treated with C_60 _fullerenes by i.p. injection**. The response is expressed as the percent of the maximal contraction induced by stimulation with a cocktail of K^+^-PSS, PGF_2α _and U-46619. Each point on the curve represents the cumulative response at each concentration of phenylephrine (PE). The data are expressed as the mean and SEM. * denotes a significant effect on E_max _compared to the control group (P < 0.05, ANOVA with unequal variance between groups).

There were no significant effects of C_60 _fullerene treatment on the EC_50 _or E_max _values of PE-induced vasoconstriction (figure 5, table 1).

## Discussion

In this study we showed that systemic treatment with C_60 _fullerenes in apoE^-/- ^mice was associated with reduced vasorelaxation responses indicating that treatment with pristine C_60 _fullerene nanoparticles alters the balance of the vasomotor functions.

Treatment with C_60 _fullerenes at a dose of 0.05 mg/kg resulted in significantly lower E_max _values of the acetylcholine-induced relaxation of aorta segments in both young and old apoE^-/- ^mice. A similar tendency was also observed for the greater dose (0.5 mg/kg), although it only reached statistical significance in the group of young apoE^-/- ^mice: this might be due to a slightly smaller variation in that group. The old male apoE^-/- ^mice were only exposed to 0.5 mg/kg of C_60 _fullerenes as part of a pilot study to assess the reliability of vasomotor function tests. The assessment of the vasomotor function in aorta rings of the arch of the vessel of the male apoE^-/- ^mice were unsatisfactory because the results were rather variable and the endothelium-dependent vasorelaxation was severely affected, although a decrease in E_max _of the endothelium-independent vasorelaxation was suggested for SNP. In addition, the results for the male mice are inconclusive because the few animals per group are associated with low statistical power. For the main experiment in female apoE^-/- ^mice we therefore used the more distal portion of the aorta for the investigation of the vasomotor function. This means that it is difficult to directly compare vasomotor response in the young and old female apoE^-/- ^mice because the aortic arch and the more distal part of the descending aorta have different vascular architecture and atherosclerotic progression. The data observed in this investigation are in keeping with our previous study on the effect of i.p. injection of diesel exhaust particles, which we found to deteriorate the endothelium-dependent acetylcholine-response, whereas the EC_50 _and E_max _values of the SNP-response could not be estimated because sigmoid concentration-response curves were not obtained [17]. This was achieved in the present study possibly by adding two low concentrations of SNP and allowing more time for each response to reach a steady state. NO is released intracellularly by metabolism of SNP and it is thus difficult to assess whether the reduced relaxation is due to interference with the metabolism or downstream signalling. Comparison with other NO donors such as diethylamine NONOate or spermine NONOate, which release NO directly, together with NO-independent vasodilators (e.g. calcium channel blocker), would be required for further understanding. In contrast to the dysfunction of the vasorelaxation, vasoconstriction does not appear to be affected by C_60 _treatment. The unaltered effect of the vasoconstriction is not likely to be due to insensitivity of the method because the order in which the segments were mounted in the myograph and used in the different concentration-response experiments was fixed with regard to distance from the heart in order to reduce the variation between experiments. It is possible that there may have been a difference in sensitivity to particle exposure along the length of the aorta, but as the segments used for the PE experiments were always dissected from a slightly more proximal part of the aorta than the segments used in the acetylcholine experiments, this should if anything have led to a higher sensitivity of the segments used in the PE experiments than in the segments used in the acetylcholine experiments. Generally, the data reported here indicate that the balance between vasorelaxation and vasoconstriction is shifted towards the latter. A direct consequence of this unbalance in the vasomotor function is increased stiffness of the vessels as has been shown in mesenteric arteriolar vessels of aged apoE^-/-^mice [22].

Oxidative stress is considered to be an important mechanism of action describing the toxic effect of particulate matter [23,24]. Biomarkers of oxidative stress, including oxidation products of biomolecules such as oxidatively modified DNA base lesions have been found in target tissue of animals and in blood cells and urine of humans as well as in in vitro models in relation to particulate matter and especially nanoparticles [25]. Particle-generated oxidative stress may arise as a consequence of direct generation of reactive oxygen species on the surface of the particles or indirectly by particle-elicited inflammatory reactions [23]. It seems unlikely that the alteration in the vascular function in our study was elicited by C_60 _fullerene-generated inflammatory reactions because the post-treatment period was only one hour and the inflammatory potential of C_60 _fullerenes appears to be modest [26]. However, we have shown that our batch of pristine C_60 _fullerenes can generate reactive oxygen species in aqueous solution as well as within cultured cells, although the concentration-response relationship was bell-shaped [27]. It is thus possible that the direct presence of C_60 _fullerenes in the vessel wall is associated with increased generation of reactive oxygen species. This would imply translocation of C_60 _fullerenes to the circulation. In a previous study on diesel exhaust particles we observed that the treated mice had particulate matter in lymph nodes of the head/neck region, which could have originated from both trapping of free particulate matter in the lymph node or migration of macrophages containing engulfed particulate matter [28]. However, most studies have focussed on the translocation of particulate matter from the lungs to the circulation and the most recent, employing impeccable methodology, indicate very little translocation of particulate matter [29-32]. Although we do not have final proof of any extent of C_60 _fullerenes translocation, our data suggest that particulate matter in the peritoneum reaches the circulation and directly affects vascular function. Our results on the endothelium-dependent vasorelaxation are in accordance with a previous investigation on *ex vivo *treatment of rabbit thoracic aorta segments with water-soluble monomalonic acid derivative of C_60 _fullerenes that was associated with a decrease in the endothelium-dependent vasorelaxation, whereas the SNP-induced endothelium-independent relaxation and PE-induced vasoconstriction was not affected in those studies [17,18]. The difference between this and our SNP-response may be due to a number of differences in the experimental setups, such as the use of ex vivo or in vivo exposure conditions, modified or pristine C_60 _fullerenes and/or the use of aorta from normal rabbits or from mice with atherosclerosis. In the studies utilizing rabbit aorta, addition of superoxide dismutase alleviated the endothelium dysfunction caused by the monomalonic derivative of C_60 _fullerenes, which indicates that the mechanism of action involves the generation of superoxide anions [17]. It is well-known that superoxide anions react with NO leading to the formation of peroxynitrite, a highly oxidizing species that forms 3-nitrotyrosine in a reaction with proteins [33]. This product was observed in aorta from apoE^-/- ^mice following exposure to air pollution particles, where the exposure was associated with increased PE-induced vasoconstriction and lower responsiveness of acetylcholine-induced vasorelaxation [8]. However, besides the generation of superoxide anions other scavenging of NO could also impair vasorelaxation, and C_60 _fullerenes may have more complicated mechanisms of action in this respect. For instance, it has been reported that hexasulfobutylated C_60 _fullerenes potentiated the endothelium-dependent vasorelaxation in aorta segments from guinea pigs [34]. In addition, it has been reported that other derivatives of C_60 _fullerenes (called C_3_- or D_3_-*tris*-malonyl-C_60_-fullerene) inhibited the activity of eNOS and C_3_-*tris*-malonyl C_60 _fullerenes, which had the largest hydrophobic surface area, inhibited eNOS the most [35]. This is an effect which could be predicted to inhibit the acetylcholine-dependent vasorelaxation of the aorta. Collectively, the data obtained so far from miscellaneous C_60 _fullerenes suggest that it is difficult to draw general conclusions based on extrapolation from a single type of particle. In addition, it is likely that the dose of C_60 _fullerenes is an important issue to consider.

Our data fit with a general picture observed following treatment with air pollution particles in mice where it has been shown that intratracheal instillation of ambient particulate matter was associated with reduced maximal acetylcholine-response and there was no change in vasoconstriction induced by PE [36]. In contrast, it has been shown that intratracheal instillation of ambient particulate matter in spontaneous hypertensive rats was associated with increased acetylcholine-dependent vasorelaxation and no effect related to PE-induced vasoconstriction [37]. It is possible that differences in the preexisting conditions and/or species can explain the discrepancy in the acetylcholine-induced vasorelaxation. In addition, long-term exposure to concentrated ambient particles was associated with altered vasomotor function in terms of both vasorelaxation and vasoconstriction, although this might partly be explained by particle-induced acceleration of atherosclerosis because the mice exposed to particulate matter exhibited a larger area of aorta covered with plaques [8]. In healthy humans, short bouts of exposure to air pollution particulate matter was associated with vascular impairment of vasorelaxation [38-42]. However, it is interesting that among patients with prior myocardial infarction, exposure to diesel exhaust did not aggravate pre-existing vasomotor dysfunction of endothelium-dependent or endothelium-independent vasorelaxation [43]. The results from our investigation calls attention to the possible detrimental effect of low doses of C_60 _fullerenes in humans considering that exposure to the C_60 _particles elicits a similar pattern of vascular motor-function effect related mainly to the endothelial cells whereas a direct effect on the smooth muscle cells is less pronounced.

The exposure concentrations of pristine C_60 _fullerenes in the environment, consumer products or drugs are still largely unknown. The highest dose of C_60 _fullerenes (0.5 mg/kg bodyweight) in the present experiment was chosen because we have previously found this dose to be associated with the largest effect in terms of endothelial dysfunction by exposure to diesel exhaust particles by the same route of exposure [16]. The maximum effect of the C_60 _fullerenes appeared to be attained already at 0.05 mg/kg in the present investigation. However, the effect was only a 15–30% reduction in the maximal endothelium-dependent relaxation and a 50–60% increase in EC_50 _of the SNP response, which might have limited impact on the risk of development of cardiovascular disease. In a recent study on inhalation exposure in rats with the cumulated dose of about 0.3 mg/kg (assuming the rats weighed 250 g), there was minimal pulmonary toxicity, whereas the C_60 _fullerene treated group showed weak signs of hepatic effects and the creatine kinase concentration in serum was increased suggesting subtle toxic effects to the myocardium [44]. We believe that the lowest dose in the present experiment (0.05 mg/kg bodyweight) is rather modest, although it is difficult to provide a reasonable dose-justification since we have no reliable information about exposure to humans. It appears that the doses of C_60 _fullerenes, which have been used in other *in vivo *studies by the same route of exposure, have been one (3.5 and 7.0 mg/kg bodyweight; [45] and two (30 and 100 mg/kg bodyweight; [21] orders of magnitude larger than the doses used in this experiment.

## Conclusion

Our study indicate that systemic exposure to C_60 _fullerenes by intraperitoneal injection at low doses can disturb the vasomotor balance toward a reduced vasorelaxation in apoE^-/- ^mice, which are hyperlipidemic animals prone to develop atherosclerotic lesions resembling those observed in humans. We therefore advise that caution should be exercised when these and similar nanomaterials are used in nanomedicine and other parental administrations.

## Methods

### Animals

The apoE^-/- ^(C57BL/6-Apoe ^tm1^) mice were purchased from Taconic MB (Ejby, Denmark) at the age of 5–8 weeks. They were housed in cages with a 12 h day-night cycle and were provided with unlimited access to standard mouse chow (Standard Altromin no.1314, Lage, Germany) and tap water. The mice were exposed to pristine C_60 _fullerenes at the age of 11–13 or 40–42 weeks. We have previously observed that the plaque area was about 1% of the total aorta in apoE^-/- ^mice at the age of 11–13 weeks [16]. In this study, we assessed the level of atherosclerosis in the aorta of two apoE^-/- ^females at the age of 40–42 weeks by means of digital microscope images on which the plaque-area was determined with a Leica imaging program IM50 (Leica Microsystems Imaging Solutions). For the main experiment we used female mice. In the first experiment we investigated the effect of C_60 _fullerenes in segments from the aortic arch of young female apoE^-/- ^mice. Subsequently, we also carried out a small pilot experiment with 40–42 weeks old male mice exposed to C_60 _fullerenes in order to assess whether or not it was possible to obtain reliable measurements of the vascular function in the aortic arch from aged apoE^-/- ^mice. Male apoE^-/- ^mice were used for this, because they were extra animals that had not been used in other experiments and were available at the time of this investigation. In addition, it has been shown that male and female apoE^-/- ^mice had similar lesion size and composition at 48 weeks of age [46]. Based on the results from the study with the old male apoE^-/- ^mice, we decided to measure the vascular function on segments obtained from the middle part of the thoracic aorta, which was less covered with atherosclerotic plaques. Institutional guidelines for animal welfare were followed and the Danish Ethical Committee for Animal Studies approved the animal experiments.

### Particles

The sample of pristine C_60 _fullerene was 99.9% pure with a declared primary particle size is 0.7 nm (Sigma Aldrich, Denmark). We have previously reported an analysis on the particle characteristics of this batch of pristine C_60 _fullerenes: the BET surface area of the preparation is less than 20 m^2^/g and it generates low amounts of reactive oxygen species in aqueous solution [27]. The particles were suspended by sonication in a solution containing 90% sterile, isotonic saline and 10% bronchoalveolar lavage fluid. The latter was prepared by flushing the lungs of unexposed apoE^-/- ^mice twice with 0.6 ml isotonic saline. The particle suspensions were sonicated on ice using a Branson Sonifier S-450D (Branson Ultrasonics Corp., Danbury, CT, USA) equipped with a disruptor horn (Model number: 101-147-037). It was operated under the following settings: total sonication time 15 min, alternating with a 55 s pulse ON and a 5 s pause and amplitude of 10%. Control solutions were prepared containing 90% isotonic saline and 10% bronchoalveolar fluid from apoE^-/- ^mice. The solutions were divided in aliquots and immediately frozen at -80°C until use. The solutions were thawed at room temperature prior to use. Analysis of the particle size in suspension showed that the majority of the C_60 _fullerenes existed as agglomerates that were larger than 1 μm in diameter; we could not detect nano-sized C_60 _fullerenes in the solution because of the agglomeration [26].

### Administration of C_60 _fullerenes

The mice were given C_60 _fullerenes by i.p. injection and were killed 1 hour later. The female mice received 0, 0.05 or 0.5 mg/kg bodyweight of C_60 _fullerenes (n = 9–11 per group), whereas only the largest dose was administrated in the pilot experiment with the old male mice (n = 5 per group). The mice were killed under general anaesthesia with Hypnorm-Dormicum by asphyxiation. The heart and aorta were carefully dissected and placed in ice cold oxygenated physiological saline solution (PSS: 119 mM NaCl, 25 mM NaHCO_3_, 4.7 mM KCL, 1.18 mM KH_2_PO_4_, 1.17 mM MgSO_4_·7H_2_O, 1.5 mM CaCl_2_·2H_2_O, 0.027 mM ethylene diamine tetraacetic acid and 5.5 mM glucose, pH = 7.4). The surrounding connective tissue was removed and the aorta was cut in segments of approximately 1.5 mm in length starting from immediately after the three large side branches of the aortic arch (young female and old male mice) or in the more distal descending aorta (old female mice).

### Vasomotor function

Three aorta segments from each mouse were mounted on steel pins with a diameter of 150 μm in the organ baths of the myograph (multi Myograph 610 M from Danish Myo Technology, Aahus, Denmark) containing 5 ml cold oxygenated PSS continuously perfused with a 95% O_2 _and 5% CO_2 _gas mixture. The order in which the aorta segments were mounted in the organ baths was randomized with regard to exposure-group but in order to reduce unnecessary variation the segments were mounted in a fixed order with regard to the specific concentration-response experiment they were used in. The segment used in the SNP experiment was the most proximal (closest to the heart), the segment distal to this was used in the phenylephrine experiment and the segment distal to this was used in the acetylcholine experiment. The myograph was connected to a computer and the data were processed by the PC-program Myodaq (Danish Myo Technology, Aarhus, Denmark). The temperature in the organ baths was slowly raised to 37°C and the segments were allowed to equilibrate for 30 min. A standard normalization procedure was performed in which the vessels were stretched to their optimal lumen diameter (*l*_1_) in order to ensure an optimal development of active tension in the aorta segments. The optimal lumen diameter was calculated by the Myodaq program from a standard curve (*l*_1 _= 0.9*l_100_) estimated by recording the tonus in the aorta segments while increasing the mechanical stretching in a stepwise manner. The value *l*_100 _is the diameter of the vessel at the physiological transmural pressure of 13.3 kPa. The vessels were subsequently allowed to equilibrate for 30 min. Next the aorta segments were contracted by substituting the PSS in the organ baths with 5 ml warm, oxygenated 125 mM K^+^-PSS (119 mM KCl, 25 mM NaHCO_3_, 4.7 mM KCL, 1.18 mM KH_2_PO_4_, 1.17 mM MgSO_4_·7H_2_O, 1.5 mM CaCl_2_·2H_2_O, 0.027 mM ethylene diamine tetraacetic acid and 5.5 mM glucose, pH = 7.4) for approximately 10 min before washing 4 times with PSS. This was repeated for a total of three times to verify viability of the aorta segments, reproducibility of contractions and to deplete the sympathetic nerve endings of neurotransmitters, noradrenaline in particular.

The endothelium-dependent vasorelaxation was assessed as acetylcholine concentration-response curves in experiments where the aorta segments had been precontracted with 1–6 μM prostaglandin F_2_α (PGF_2_α, Dinolytic vet. 5 mg/ml from Pharmacia NV/SA, Puurs, Belgien) before adding increasing concentrations of acetylcholine (Sigma-Aldrich Chemie Gmbh, Schelldorf, Germany) in a stepwise manner from 0.1 nM – 0.01 mM. The endothelium-independent vasorelaxation was tested by adding increasing concentrations of the NO donor SNP (0.1 nM – 0.01 mM, Sigma-Aldrich Chemie Gmbh, Schelldorf, Germany) to PGF_2_α precontracted aorta segments. SNP has been widely used for this purpose particularly in the clinical setting and in whole animal preparations, whereas the use of SNP for ex vivo investigation of endothelial-independent has been less extensive because of the opportunity to use other types of NO donors. Other segments were used for the assessment of PE-induced vasoconstriction. The concentration-response curves were generated by increasing the concentration of PE (Sigma-Aldrich Chemie Gmbh, Schelldorf, Germany) in the organ baths in a stepwise manner (0.1 nM – 0.1 mM). The segments exposed to acetylcholine or PE were then contracted once with 125 mM K^+^-PSS followed by 4 PSS rinses, after which an endothelium check was performed by precontracting the vessels with approximately 3 μM PGF_2_α before adding 0.01 mM acetylcholine. Finally the aorta segments were stimulated with a cocktail of 125 mM K^+^-PSS, 0.01 mM PGF_2_α and 1 μM U-46619 (Cayman Chemicals, Ann Arbor, USA) to induce a maximal contraction to which any other contraction could be held in comparison.

### Curve fitting and analysis

All steps in the concentration-response curves were recorded at the point considered to be the lowest (acetylcholine and SNP) or highest (PE and cocktail) steady state value obtained at that concentration of vasoactive reagent. The basal tone of the aorta was subtracted from all recordings of drug-induced vessel tone, estimated by placing a baseline in the Myodaq program. The relaxation caused by acetylcholine and SNP was expressed as the percent relaxation of the precontraction tension produced by PGF_2α_. The contraction caused by PE was expressed as the percent of the maximal contraction obtained when stimulating the aorta segment with a cocktail consisting of K^+^-PSS, PGF_2α _and U46619. The EC_50 _and E_max _values were calculated using the GraphPad Prism version 4 (San Diego, USA). The data were fitted to sigmoid curves with varying slopes using non-linear regression according to the following equation:

Y = Bottom + (Top - Bottom)/(1 + 10 ^ ((Log EC_50 _- X) * HillSlope)

X is the logarithm of concentration and Y is the response. Y starts at Bottom and goes to Top with a sigmoid shape. We analyzed the data by ANOVA test with unequal variance, because there was not homogeneity of variance between the groups. The curve fitting was carried out in Graph Pad Prism 4 and the E_max _and EC_50 _with corresponding 95% confidence intervals (CI) were calculated. Groups with non-overlapping 95% CI are considered statistically significant different at 5% level. The data points on each curve are expressed as mean ± SEM.

## Abbreviations

apoE: apolipoprotein E; ACh: acetylcholine; CI: confidence interval; eNOS: endothelial nitric oxide synthetase; NO: nitric oxide; PE: phenylephrine; PSS: physiological saline solution; SEM: standard error of the mean; SNP: sodium nitroprusside.

## Competing interests

The authors declare that they have no competing interests.

## Authors' contributions

HWA, SL and PM conceived the study. LKV, HWA, SL and PM designed the experiments. LKV and JKF made the experiments on vasomotor function, assisted and supervised by MS. The characterization of the particulate matter and supervision of the animal experiments were carried out by NRC. LKV made the draft of the manuscript, which was revised critically by SL and PM. All authors have read and approved the manuscript.
